# Supplementary figures and images for: Sampling through time and phylodynamic inference with coalescent and birth–death models
Source: J R Soc Interface. 2014 Dec 6;11(101):20140945. doi: 10.1098/rsif.2014.0945 (PMC4223917; doi:10.1098/rsif.2014.0945)

# Estimated birth rate

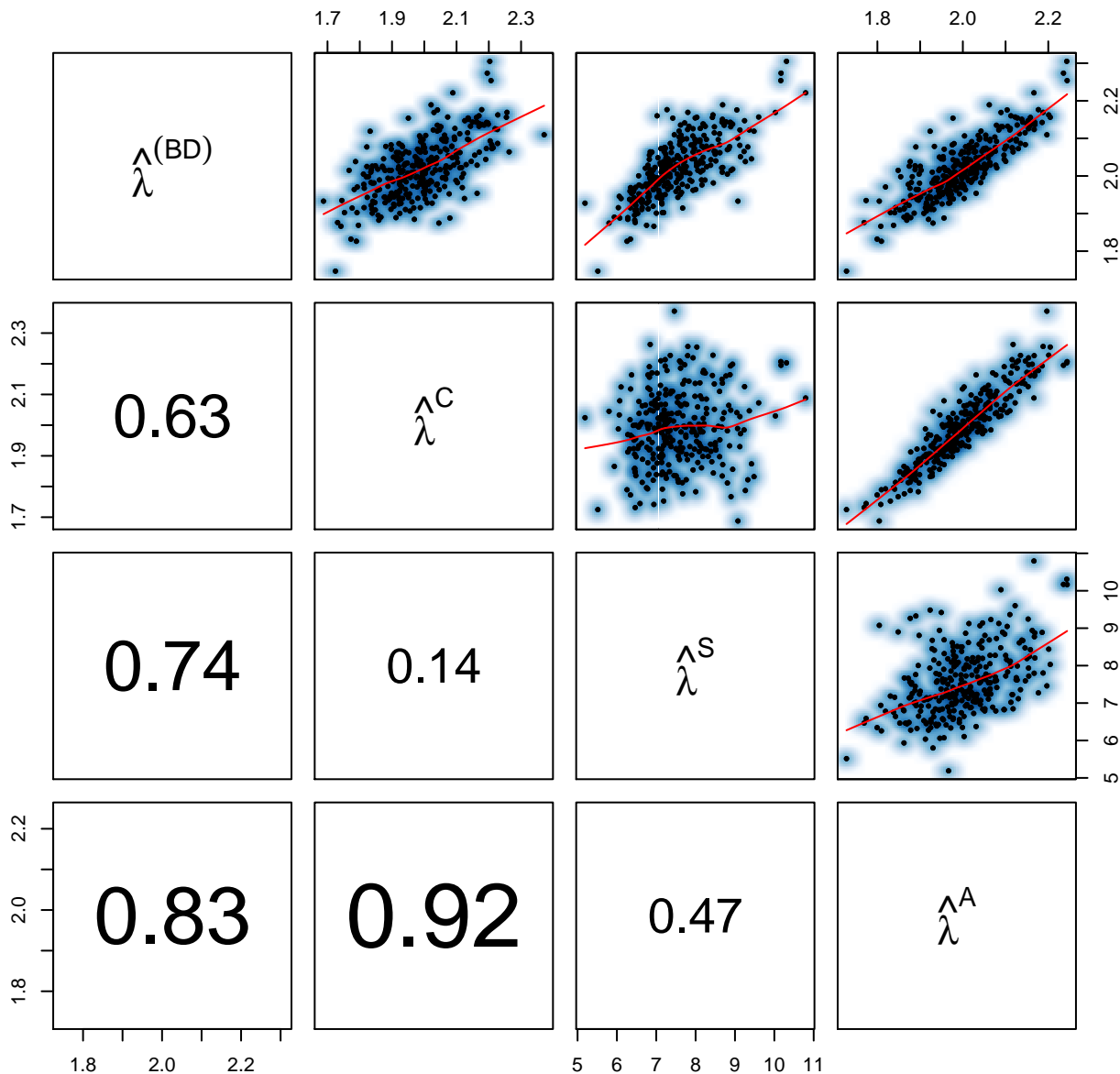

Supplement: Figure S1. [file rsif20140945supp1.pdf]

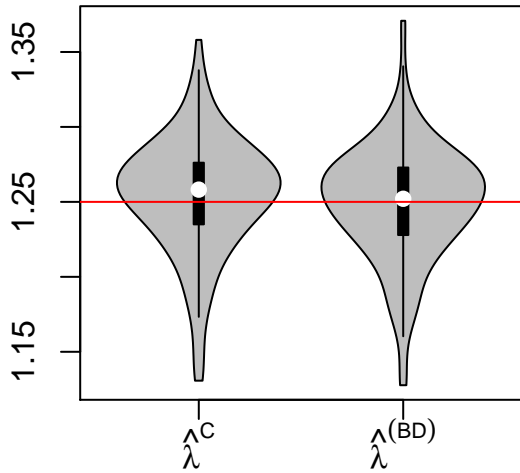

BDM log likelihood

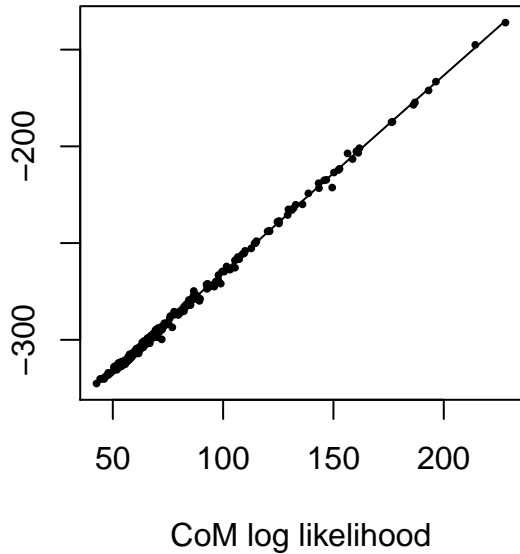

Supplement: Figure S2. [file rsif20140945supp2.pdf]

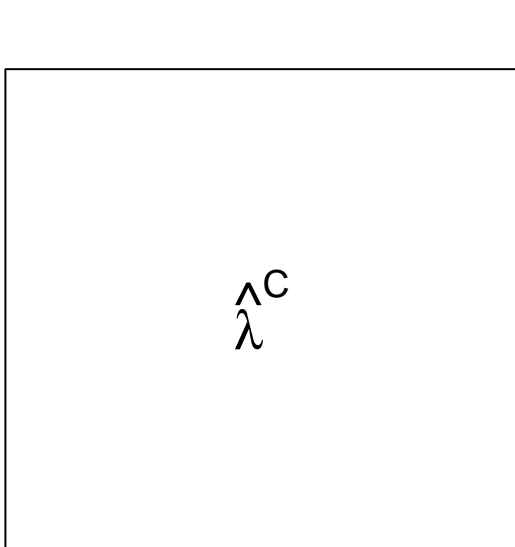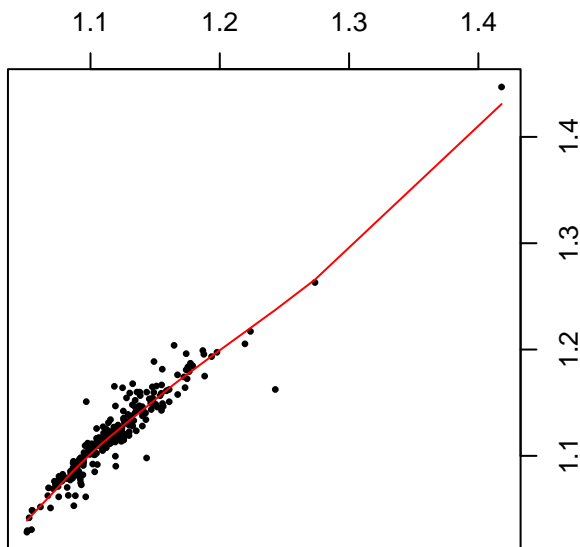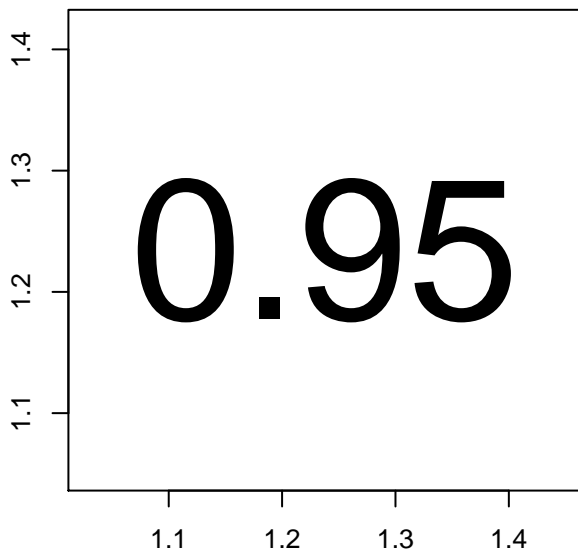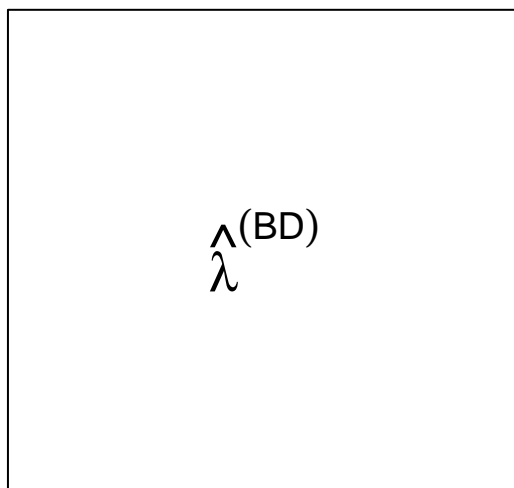

Supplement: Figure S3. [file rsif20140945supp3.pdf]
